# Phases I–III Clinical Trials Using Adult Stem Cells

**DOI:** 10.4061/2010/604713

**Published:** 2011-02-23

**Authors:** Jeffrey M. Gimble, Bruce A. Bunnell, Louis Casteilla, Jin Sup Jung, Kotaro Yoshimura

**Affiliations:** ^1^Stem Cell Biology Laboratory, Pennington Biomedical Research Center, Louisiana State University System, Baton Rouge, LA 70808, USA; ^2^Center for Stem Cell Research and Regenerative Medicine, Tulane University School of Medicine, New Orleans, LA 70808, USA; ^3^CNRS, UMR 5273, STROMALab, BP 84225, 31432 Toulouse, France; ^4^Université de Toulouse, UPS, UMR 5273, STROMALab, BP 84225, 31432 Toulouse Cedex 4, France; ^5^Inserm, U1031, STROMALab, BP 84225, 31432 Toulouse Cedex 4, France; ^6^Laboratory for Cell Therapy and Tissue Regeneration, Department of Physiology, School of Medicine, Pusan National University, Busan 626-870, Republic of Korea; ^7^Department of Plastic Surgery Graduate School of Medicine, The University of Tokyo, Tokyo 113-8655, Japan

Mesenchymal progenitor/stromal/stem cell (MSC) research has made substantial progress during the past decades. Investigators have published a wealth of basic science and preclinical data documenting the potential utility, efficacy, and safety of MSCs. The majority of this work has focused on the bone marrow- or adipose-derived MSCs. Since this body of evidence has set the stage for clinicians to advance from the bench to the bedside, *Stem Cells International* set out to publish a special issue devoted to the topic of *Phases I–III Clinical Trials Using Adult Stem Cells*. The result is a collection of ten outstanding articles submitted by investigators representing ten countries across Asia, Europe, and North America. In all cases, the methods section of each manuscript included a statement documenting that the clinical investigations were performed following institutional review board approval and/or that patient informed consent had been provided prior to the conduct of the study. 

Bieback et al. in Germany (*Translating research into clinical scale manufacturing of mesenchymal stromal cells*) and B. Philippe et al. in France (in “*Culture and Use of Mesenchymal Stromal Cells in Phase I and II Clinical Trials”*) have focused on the process of MSC isolation and expansion with respect to current Good Manufacturing Practices (cGMP). These authors highlight the state of the art as well as the challenges facing the development of clinical grade cells for therapeutic applications. 

From a regulatory perspective, the simplest application of MSC is to use them in the context of their tissue of origin. S. Akita et al. in Japan (in “*Noncultured Autologous Adipose-Derived Stem Cell Therapy for Chronic Radiation Injury”*) have exploited adipose-derived stem cells for soft tissue regeneration. They describe their clinical experience using autologous adipose-derived stem cells in combination with growth factors and scaffold to treat ten patients suffering from dermal radiation lesions. A series of three review papers from Belgium (“*Phase 1–3 Clinical Trials Using Adult Stem Cells in Osteonecrosis and Nonunion Fractures*” by J. –P. Hauzeur and V. Gangji), the USA and China (“*Mesenchymal Progenitor Cells and Their Orthopedic Applications: Forging a Path towards Clinical Trials*” by D. S. Shenaq et al.), and the Netherlands (“*Clinical Applications of Human Mesenchymal Stromal Cells for Bone Tissue Engineering*” by Chatterjea et al.) have examined the use of bone marrow-derived MSC for orthopedic applications. Each work provides a unique perspective on the published clinical trials using MSC to treat cartilage and tendon repair, critical sized defects, metabolic bone diseases, nonunion fractures, and/or osteonecrosis. 

From a public health perspective, MSC applications for common cardiac and central nervous system disorders could have substantial health and societal benefits. R. Sanz-Ruiz et al. in Spain (in “*Phases I–III Clinical Trials Using Adult Stem Cells”*) have emphasized the critical importance of randomized clinical trials to assess the utility of MSCs as they focus on the clinical evidence supporting MSC-based treatment of cardiac disease. Furthermore, they review the guidance documents on future clinical trials provided by a task force convened by the European Society for Cardiology. P. A. Walker et al. in the USA (in “*Progenitor Cell Therapy for The Treatment of Central Nervous System Injury: A Review of the State of Current Clinical Trials*) provide a similar perspective relating to central nervous system disorders. They evaluate the use of bone marrow-derived MSCs for ischemic stroke, spinal cord injury, and traumatic brain injury and conclude that there remains a need for further evidence to support these clinical applications.

There are potential MSC applications for metabolic disorders as well. A. C. Piscaglia et al. in Italy (in “*Stem Cell Therapies for Liver Disease: State of the Art and New Perspectives*) have reviewed the limited number of clinical trials using hematopoietic stem cell (HSC), MSC infusion, or cytokine (G-CSF) therapy to reverse hepatic injury due to cirrhosis, drug exposure, or other causes of end stage liver disease. Likewise, A. V. Vanikar et al. from India (in “*Cotransplantation of Adipose Tissue-Derived Insulin Secreting Mesenchymal Stem Cells A Novel Therapy for Insulin-Dependent Diabetes Mellitus”*) describe their experience combining adipose- and bone marrow-derived MSC to treat a cohort of 11 diabetic subjects for a period of up to 23 months. They report improved hemoglobin A1C levels as well as decreased daily requirements for exogenous insulin.

Uniformly, these authors highlight both the promise and the challenges faced by this emerging field of medicine. Their manuscripts identify the critical need for additional prospective, randomized controlled clinical trials evaluating all cell-based therapies, regardless of the underlying disorder. In summary, this *special issue *provides a snapshot of the current status of MSC-based clinical trials across the globe. Hopefully, this publication will provide a benchmark for future meta-analyses evaluating a far greater body of clinical evidence regarding the safety and efficacy of MSC therapies.

*Jeffrey M. Gimble* *Jeffrey M. Gimble* *Bruce A. Bunnell* *Bruce A. Bunnell* *Louis Casteilla* *Louis Casteilla* *Jin Sup Jung* *Jin Sup Jung* *Kotaro Yoshimura* *Kotaro Yoshimura*

